# Selection Indicates Preference in Diverse Habitats: A Ground-Nesting Bird (*Charadrius melodus*) Using Reservoir Shoreline

**DOI:** 10.1371/journal.pone.0030347

**Published:** 2012-01-27

**Authors:** Michael J. Anteau, Mark H. Sherfy, Mark T. Wiltermuth

**Affiliations:** Northern Prairie Wildlife Research Center, U.S. Geological Survey, Jamestown, North Dakota, United States of America; Dalhousie University, Canada

## Abstract

Animals use proximate cues to select resources that maximize individual fitness. When animals have a diverse array of available habitats, those selected could give insights into true habitat preferences. Since the construction of the Garrison Dam on the Missouri River in North Dakota, Lake Sakakawea (SAK) has become an important breeding area for federally threatened piping plovers (*Charadrius melodus*; hereafter plovers). We used conditional logistic regression to examine nest-site selection at fine scales (1, 3, and 10 m) during summers 2006–2009 by comparing characteristics at 351 nests to those of 668 random sites within nesting territories. Plovers selected sites (1 m^2^) that were lower than unused random sites, increasing the risk of nest inundation. Plovers selected nest sites that were flat, had little silt, and at least 1 cobble; they also selected for 3-m radius nest areas that were relatively flat and devoid of vegetation and litter. Ninety percent of nests had <38% coverage of silt and <10% slope at the site, and <15% coverage of vegetation or litter and <31% slope within the 3-m radius. Gravel was selected for at nest sites (11% median), but against in the area 10-m from the nest, suggesting plovers select for patches or strips of gravel. Although elevation is rarely evaluated in studies of ground-nesting birds, our results underscore its importance in habitat-selection studies. Relative to where plovers historically nested, habitat at SAK has more diverse topography, substrate composition, vegetation communities, and greater water-level fluctuations. Accordingly, our results provide an example of how habitat-selection results can be interpreted as habitat preferences because they are not influenced by desired habitats being scarce or absent. Further, our results will be useful for directing habitat conservation for plovers and interpreting other habitat-selection studies.

## Introduction

Understanding habitat preferences of animals is important because they represent proximate cues for selecting resources that maximize individual fitness [1], [2], [3], [4]. However, often correlations between cues and the ultimate mechanism that influences fitness are intermediated by other factors which makes those correlations susceptible to change in altered or managed environments [3]. Given that land use changes have affected the composition and availability of habitat for many species around the globe [5] cues animals evolved may not be suitable for maximizing fitness, so understanding habitat preferences is the first step to evaluating habitat suitability [3]. Furthermore, understanding habitat preferences is an important step for the conservation and management of species of concern because effective management must target cues that lead to sites being used and mechanisms that maximize fitness.

Unfortunately, despite substantial developments in quantitative techniques for evaluating selection [6], [7], [8], habitat preferences are inherently difficult to evaluate in nature because each area provides differing availability of various habitat features from which individuals must choose [6], [9]. Reviews of many individual studies can provide inference for habitat preferences if those studies represent available habitat in the area of interest. However, a recent review of a highly studied subject, duck food preferences, indicated that there is uncertainty over resource preferences because many of the past studies are poorly replicated and did not relate data on resources selected with those available [10]. Landscape-scale studies that randomly sample hundreds of individuals at hundreds of sites may provide rigorous inference to resource preference within the landscape sampled [11], [12]; however, individuals often do not have all available habitats to choose from within each site and desired but rare or absent resources would therefore be under represented. A more rigorous examination of preference would involve experimentally controlling density of animals while providing all potentially available habitats in one discrete area and observing what habitat is selected. Unfortunately, conducting such a prospective experiment in nature generally is not feasible. As an alternative, we present an example of a retrospective evaluation of nest-site selection for a ground-nesting bird using a novel environment; a diverse array of habitat features present in all other breeding areas where this species has previously been observed were available throughout our study area. Accordingly, we argue that habitat selection under these circumstances can provide inference for habitat preference.

While animals select habitat or resources in many aspects of their life-history, nest-site selection can be particularly important for the fitness of birds because once a site is selected it must remain a suitable location for the duration of laying, incubation, and brood rearing (in the case of altricial birds) [1], [13]. There are a number of threats to nest survival that likely have shaped nest-site selection traits throughout the evolution of ground-nesting birds. The major threats include 1) extreme weather events (heat, cold, high winds, hail); 2) inundation from pulses in river flows, storm surges, large tide cycles, or from pooling of local precipitation; and 3) nest predation [14], [15] (M. J. Anteau, unpublished data). There are two main groups of animals that depredate nests on the ground, avian predators that mainly forage visually and mammalian predators that forage by scent and use some visual cues [4]. Thus, there is a selection pressure for incubating adults and nests to be 1) protected from extreme weather, 2) on high or well drained areas, protected from inundation, and 3) cryptic to reduce detection by visual predators.

Piping plovers (*Charadrius melodus*; hereafter plovers) are a federally (United States) listed bird that evolved using nesting habitats on coastal beaches, alkali wetlands, and riverine sandbars that generally are devegetated, flat, sandy areas, with occasional scattered pebbles or gravel [14], [15], [16], [17]. Since the 1950s, with the construction of dams on large river systems in the Northern Great Plains, plovers began nesting on shorelines and islands of reservoirs [17]. In 2005, 64% of Missouri River plovers were observed on reservoir habitats (US Army Corps of Engineers [USACE], unpublished data), and 29% of the Northern Great Plains population used reservoir habitat during summer 2006 [18]. Potential nesting habitats on reservoirs consist of a wider variety of substrate sizes, elevations, and slopes, than those present on coastal shorelines, alkaline lakeshores, or riverine sandbars (see Discussion).

Understanding habitat selection, particularly elevation and spatial needs, is critical to evaluating habitat suitability in reservoir systems because relative to natural or historic habitats reservoirs have large variations in 1) elevation of potential nesting habitat, 2) intra-annual water levels, and 3) sizes of potential habitat patches. Furthermore, examining nest-site selection on reservoirs should provide inference into habitat preferences of plovers because a wide array of potential habitats is available, including areas with habitat similar to that in other principal landforms. Accordingly, in 2006–2009 we evaluated characteristics that influence nest-site selection by plovers at Lake Sakakawea (hereafter SAK), a reservoir and important breeding area. We examined habitat characteristics at 349 nests and 668 unused random sites, as well as within 3-m and 10-m radiuses from nest and random sites to determine whether plovers use fine-scale features to select nesting habitats and to identify the minimum area which plovers use to select habitats.

## Results

At SAK, we found and measured habitat characteristics at 52, 103, 83, and 111 nest sites in 2006–2009, respectively. Nest searches were conducted on segments at a mean interval of 4.7, 2.9, 2.9, and 3.1 days in 2006–2009, respectively. The mean number of days between discovery of the nest and estimated nest initiation was 7, 3.8, 3.3, and 2.7 days in 2006–2009, respectively.

The probability that a site, within a territory, was used for nesting decreased when sites had greater percent coverage of silt and percent bare-substrate obstruction (i.e., vegetation and litter cover) 3 m from the site, but sites with at least one cobble present had greater probability of use than those that did not have any cobble (Table 1). The probability that a site was used for nesting decreased as the elevation, relative to water level at nest initiation, increased. While greater percent coverage of gravel increased the probability of a site being used for nesting, greater coverage of gravel 10 m from the site decreased that probability. Greater percent slopes at the site and within 3 m of the site decreased the probability that a site was used for nesting. However, slope at the site appeared to be a more important factor driving selection than that at 3 m because slope at the site had a greater standardized odds ratio than that at 3 m (Table 1). Based on standardized odds ratios, effect sizes of variables from highest to lowest were as follows: 1) presence of a cobble at the site, 2) bare-substrate obstruction at 3 m, 3) percent silt at the site, 4) slope at the site, 5) relative elevation of the site, 6) percent gravel at the site, 7) slope within 3 m of the site, and 8) percent gravel 10 m from the site. We report medians and 10^th^ and 90^th^ percentiles at nest and random sites for each of these variables (Table 2).

**Table 1:** Model averaged parameter estimates, standard errors (SE), lower 95% confidence limits (LCL), upper 95% confidence limits (UCL), and standardized odds ratios for variables from 20 candidate models we used to examine potential influences on nest-site selection of Piping Plovers at Lake Sakakawea.

Variable	Estimate	SE	LCL	UCL	Odds Ratio
**SILT** a	**−0.019**	**0.005**	**−0.028**	**−0.009**	**1.841**
PEB^b^_S^c^	0.000	0.000	0.000	0.000	n/a
**GRAV** d **_S**	**0.038**	**0.008**	**0.022**	**0.054**	**1.447**
**COB** e	**1.570**	**0.228**	**1.114**	**2.026**	**4.808**
VEG^f^_S	0.000	0.000	0.000	0.000	n/a
DIST^g^	0.004	0.004	−0.004	0.013	n/a
**RELEV** h	**−0.283**	**0.119**	**−0.521**	**−0.046**	**1.762**
**SLOPE**	**−0.082**	**0.021**	**−0.124**	**−0.041**	**1.839**
PEB_3^i^	0.000	0.000	0.000	0.000	n/a
GRAV_3	0.000	0.000	0.000	0.000	n/a
**VEG_3**	**−0.047**	**0.009**	**−0.065**	**−0.030**	**2.269**
**SLOPE_3**	**−0.017**	**0.008**	**−0.033**	**−0.001**	**1.375**
PEB_10^j^	0.000	0.000	0.000	0.000	n/a
**GRAV_10**	**−0.040**	**0.012**	**−0.065**	**−0.016**	**1.295**
VEG_10	0.000	0.000	0.000	0.000	n/a
SLOPE_10	0.000	0.000	0.000	0.000	n/a

Variables with 95% confidence limits that do not overlap 0 are depicted in bold.

a Percent coverage of silt in substrate at site.

b Percent coverage of pebble in substrate.

c Site measurement.

d Percent coverage of gravel in substrate.

e Presence or absence of a cobble.

f Percent bare substrate obstruction (vegetation+leaf litter+small debris).

g Distance (m) to shoreline of Lake Sakakawea.

h Relative elevation (m) of the nest above the pool level at initiation.

i Mean of 4 measurements taken 3 m from the site.

j Mean of 4 measurements taken 10 m from the site.

**Table 2:** Median and 10^th^ and 90^th^ percentiles of nest and random sites for variables that influence nest-site selection of Piping Plovers on Lake Sakakawea.

Variable	Random Sites			Nest Sites		
	10^th^	Median	90^th^	10^th^	Median	90^th^
SILT^a^	0	0	85.0	0	0	38.2
GRAV_S^b^	0	0	26.7	2.5	11.4	38.6
COB^c^	0	0	1	0	1	1
RELEV^d^	0.7	1.8	5.3	0.5	1.7	4.8
SLOPE^e^	0.6	4.9	16.1	0.3	3.6	9.8
VEG_3^f^	2.5	7.5	46.1	2.5	5	14.4
SLOPE_3^g^	4.5	15.5	41.5	3.9	13.1	31.1
GRAV_10^h^	0	2.5	18.8	1.1	2.8	21.5

a Percent coverage of silt in substrate at site.

b Percent coverage of gravel in substrate at site.

c Presence or absence of a cobble.

d Relative elevation of the nest above the pool level at initiation.

e Slope within 1 m of the site.

f Percent bare substrate obstruction (vegetation+leaf litter+small debris).

g Slope within 3 m of the site.

h Percent coverage of gravel in substrate 10 m from the site.

## Discussion

### Habitat Selection in a Diverse Breeding Habitat

Based on a review of published results, our study area did indeed contain a more diverse array of habitat features than other breeding habitats where this species has been observed previously. Available plover nesting habitat on the Atlantic coast had a mean slope of 8.3% (SD = 6.8 [19]), which is much lower and less variable than we observed on SAK (Min = 1%, Max = 317%, Mean = 49%, SD = 41). Although slope and elevation data are lacking from studies in other habitats, it is clear that the range of elevations in available nesting habitat observed in this study (11 m) is much larger than that on river sandbar (generally <2 m; M. J. Anteau, M. H. Sherfy, personal observations), alkali lakes (generally <1 m; K. Brennan and C. Mueller, personal communication), or coastal beaches (generally <3 m [19]). Coarse substrate (e.g., gravel and cobble) was more variable and available on SAK than on Missouri River sandbars or Atlantic beaches (Table 3). Similarly, areas of habitat with varying degrees of vegetation coverage and patch sizes were more abundant on SAK than on Missouri River sandbars or Atlantic beaches (Table 3). Accordingly, our habitat selection results can be interpreted as habitat preferences because they should not be influenced by certain habitat types being scarce or absent and each individual characteristic we found important also is represented in other breeding areas.

**Table 3:** Mean and standard deviation (SD) and 25^th^, 50^th^, 75^th^, 90^th^, and 100^th^ percentiles for variables of available Piping Plover nesting habitat at Lake Sakakawea (SAK), sandbars of the Missouri River (MR), and the Atlantic Coast (AC).

Variable^a^	Location	Mean	SD	25^th^	50^th^	75^th^	90^th^	100^th^
SILT	SAK	20	32	0	0	33	85	100
	MR^b^	19	31	0	0	23	85	85
PEBBLE	SAK	15	18	3	8	22	39	97
	MR	11	20	0	0	10	38	85
GRAVEL	SAK	9	15	0	3	10	27	92
	MR	1	3	0	0	0	3	23
COBBLE	SAK	3	8	0	0	2	9	85
	MR	0	1	0	0	0	0	10
PEB-COB^c^	SAK	26	28	4	13	43	73	100
	MR	12	21	0	0	13	48	95
	AC^d^	6	2	e	e	e	e	47
VEGTATION	SAK	12	17	4	5	13	28	100
	MR	6	12	0	0	23	85	85
	AC	4	2	e	e	e	e	49

a Percent coverage.

b Sherfy et al. 2012.

c Percent coverage of pebble, gravel, and cobble.

d Cohen et al. 2008.

e Not reported.

Examining inferred nest-site preferences from our study leads to a new and clearer understanding of habitat selection in this species and will be informative for interpreting nest-site selection studies in other habitats. On all landforms used by plovers for nesting, fluctuations in water levels typically maintain areas devoid of vegetation, but on reservoirs water-level fluctuations generally are much greater than those on other habitats. Plovers did prefer nesting on flat areas, but they nested at areas with greater slopes than expected; they also preferred lower-elevation areas, which put nests at risk of inundation (M. J. Anteau, unpublished data). Although elevation is rarely evaluated in studies of ground-nesting birds, our results underscore its importance in habitat-selection studies, particularly for waterbirds. By examining selection of habitat at 3 fine scales, we found that plovers preferred patches 6 to 20 m in diameter that were relatively flat, gravelly, and devoid of vegetation. Despite selecting for pebbles on riverine sites (M. H. Sherfy and M. J. Anteau, unpublished data) and using them to line nests, plovers did not exhibit preference for sites with pebbles at the diverse habitat. Accordingly, it appears that plovers preferred patches of gravel over pebbles.

### Slope and Elevation

Slope at nest sites of plovers has been largely unstudied, but it was generally thought that plovers selected large, flat areas for nesting [15], [19]. Golden plovers (*Pluvialis apricaria*) in the United Kingdom did select flatter sites than what was available [20]. Mean slopes at 10-m nest-areas of golden plovers were below 1.7% at two different sites, while the means for random points were 5.2 and 12.3%. At SAK, plovers appear to select flat areas 1 m around and within 3 m from their nests, which is consistent with expectations. However, habitat at SAK is topographically more diverse than that of coastal beaches, riverine sandbars, or alkali wetlands and we observed that 10% of plover nests had slopes greater than 9.8 and 31.1% at 1 and 3 m, respectively (Table 2). Moreover, mean slopes for the 3-m nest-area of plovers and random sites at SAK were markedly greater than those observed for golden plovers in the United Kingdom. Selection by plovers for flat areas for nesting indeed was stronger at 1 m around the nest than it was within 3 m from the nest. Accordingly, our results do support the idea that plovers select flatter nesting areas, but they appear more adaptable to areas with higher slopes than previously thought as long as there are smaller flat areas for nest sites.

Presumably plovers evolved nest-site selection traits on alkali wetlands, riverine sandbars, and coastal beaches, where water level generally does not vary as much as it does on reservoirs. During 2006–2009, water level at SAK increased 2.4 m, on average, between 15 May and 30 June [21]; these times generally correspond to laying and incubating periods for plovers at SAK. Our data indicated that plovers selected sites that had lower relative elevation than random sites, after controlling for all other important selection variables. The average increase in water level was greater than the median relative elevation of nests (Table 2); indeed, nest survival was particularly low for plovers at SAK due to the high likelihood of nest inundation (M. J. Anteau, unpublished data). Coupling our relative elevation and slope findings suggests that plovers favor flat, low-elevation sites for nesting. This trait is seemingly advantageous in the plover's native habitat, but is risky on reservoirs and suggests that reservoirs where water level increases during the nesting period are an ecological trap for plovers (M. J. Anteau, unpublished data).

### Vegetation and Litter

Our data indicate that plovers are tolerant to sparse vegetation (<50% cover) at the nest site, but select for bare 3-m nest areas. Similar to our findings, nest sites of plovers at Cape Cod, Massachusetts were more likely to have vegetation present than random points, but had lower amounts of vegetation farther away from nests [19]. This selection could facilitate a plover's ability to see approaching predators while keeping the nest site visually obscured by small amounts of vegetation. Indeed, during nesting and brood rearing plovers use a broken-wing display to lure scent-foraging predators away from their nest or brood [17]. Clearly, the broken-wing display is only effective if the plover can detect approaching predators. If vegetation is close to the nest and has little structure at the height of an incubating plover's eye, it would not likely obscure horizontal vision because slight movements of the plover's head would increase its view. Further, if outward vision is not appreciably obstructed, then plovers may gain some advantage to nesting in a small patch of sparse vegetation through in increase in crypsis [19] or shelter from wind or direct sunlight [22]. In contrast, Cohen et al. [23] speculated that plovers selected vegetated areas in New York because they were more secure from inundation than unvegetated areas. Unlike previous findings, our analysis does not confound effects of vegetation and elevation because we controlled relative elevation and areas that are sparsely vegetated were not coincident or correlated with elevation on SAK. Considering results from other studies in the context of our findings, plovers may tolerate small amounts of patchy vegetation at the nest site, but prefer bare areas farther out from the nest.

### Substrate

Our results indicated that plovers selected against nesting in areas with higher percentages of silt; these findings are similar to two other recent studies on nest-site selection of plovers and least terns (*Sternula antillarum*) on sandbars of the Missouri River [24] (M. H. Sherfy and M. J. Anteau, unpublished data). It is possible that this correlation is driven by a direct mechanism; for example, an avoidance of areas that may hold water or moisture on eggs. Adherence of silt to eggs was implicated as a potential cause of nest failure for least terns nesting on dredge spoil [25], [26]. However, plovers line their nest bowls with pebbles [17], [27], which would reduce egg contact with silt. Alternatively, avoidance of silt may be because it accumulates in low areas due to alluvial processes. As speculated fro least terns, perhaps plovers use silt as a cue for areas that are prone to inundation or surface water accumulation after local precipitation events [24].

On riverine sandbar habitats small pebbles are associated with the selection of nest sites (M. H. Sherfy and M. J. Anteau, unpublished data). Plovers line their nest scrapes with small pebbles, usually prior to laying eggs [27]. However, small pebbles were not important in explaining nest-site selection on SAK. Apparently, on SAK there are adequate sources of small pebbles that plovers can line their nest scrapes without noticeably selecting for small pebbles. Gravel is very rare on natural riverine sandbar habitat of the upper Missouri River [24] but not on SAK. Our findings that plovers select for gravel but not pebbles at nest sites suggest that, given the choice, they prefer gravel habitats for nest sites. Indeed, nests situated in gravel are very cryptic and potentially secure from detection by visually foraging predators because plover eggs are of similar size and shape as gravel.

Our results further suggest that plovers at SAK select gravel arranged in strips for nesting. Generally, there are large expanses of gravel available on SAK, but our data indicate that gravel strips or small patches are selected over these areas because we observed a negative correlation between probability of nesting and gravel 10 m from the nest. Generally, larger patches are more secure for ground nesting birds because predator effort is “diluted” in larger patches [28]; however, it seems unlikely that potential nest predators of plovers identify and search gravel patches. Alternatively, gravel strips on lake shorelines often have slightly higher elevation than the surrounding landscape. Plover nests often occur near the edge of these strips, slightly lower than the elevation at the center of the gravel strip. We speculate that this slight elevation variation might help conceal the body of an incubating plover while still allowing that plover to see potential predators approaching.

It is possible that selection for larger substrate types is driven by the aversion to smaller substrates like sand that are more mobile in high winds. Drifting sand can cover nests and is a potential source of nest mortality [29], [30]. However, avoidance of drifting sand does not explain the preference for gravel and certainly is not consistent with the selection of small gravel patches or strips.

We observed a selection for at least 1 cobble present at nest sites. These findings are consistent with those of previous studies from riverine sandbars, Atlantic beaches, and another inland reservoir [19], [31], [32] (M. H. Sherfy and M. J. Anteau, unpublished data). Cobbles generally are similar in size to an adult bird and when scattered on a large flat area, they likely make it more difficult for visual predators to quickly scan for adults attending nests. Similar to vegetation and debris [14], [19], cobbles may also provide some shelter from high winds.

### Implications for Conservation

Land use changes have affected the composition and availability of habitat around the globe, as a result numerous species are in jeopardy of extinction [5]. For example, availability of quality habitat within the Northern Great Plains is critical to the conservation of this threatened plover population [33], [34]. Dynamic fluctuation of Missouri River flows is necessary to create and maintain natural sandbar habitat and no longer exists, requiring alternative approaches to provide those habitats. As a result, it has become important to create and maintain habitat in alternative ways. The USACE currently funds a multi-million dollar program to mechanically create nesting habitat for least terns and piping plovers along the Missouri River system [16]. Accordingly, understanding habitat preferences is important when habitat management creates conditions that differ from those in the naturally regulated system.

When habitats change from those under which a species evolved it becomes increasingly important to understand resource-selection cues because they may no longer maximize an individual's fitness in changed habitat [3], [35]. Furthermore, if habitat cues are understood, conservation efforts could use knowledge of habitat preferences to help direct habitat selection to safer areas within a changed habitat [36]. Because our results are indicative of habitat preferences they could be useful in interpreting the results of nest-site selection studies for plovers at other locations and are a critical step toward inventory and management of habitat for this species. For example, because plovers are prone to nesting in areas that will be inundated prior to hatching in some manipulated habitats [27], [35], managers could remove gravel and cobble from those sites and supplement gravel and cobble on sites more secure from inundation. Further, gravel and cobble are relatively rare on sandbars of the Missouri River, but they could be increased on areas of mechanically created sandbars that are less likely to be inundated during the nesting season. However, we suggest that effectiveness of any management scheme to enhance plover habitat be thoroughly evaluated through an adaptive resource management framework, especially if it involves changing habitat availability of any characteristic beyond what is typically observed in nature.

Within-territory, nest-site selection at SAK can provide guidance for quantifying and managing plover habitat on SAK and likely elsewhere. Our results indicate that plovers select relatively flat areas that are well drained, devoid of vegetation, with abundant gravel, and at least 1 cobble. These areas were at least 6 m in diameter, but our findings suggest they could be less than 20 m. The 90^th^ percentile value for nests should make a suitable threshold goal for characteristics that have a negative influence on nest-site selection. For example, nesting habitat can be defined as areas that have <38% silt and <10% slope at the nest site, and <15% coverage of vegetation or litter and <31% slope in an area 3 m out from a potential nest site. Gravel positively influenced nest-site selection, but is relatively rare at most habitats; using the 90^th^ percentile as a goal for gravel would set a standard that is rarely observed. We suggest that the mean value of gravel for nest sites (18%) would make a suitable goal for a minimum level. Levels for slope and bare substrate obstruction that we define here will be useful in defining nesting habitat for the purpose of remotely sensing and quantifying the amount of habitat available to plovers at SAK and elsewhere. Furthermore, our results will be useful to set goals for characteristics of created or managed habitat that is likely to be used by plovers. However, factors that influence selection of territory sites also are clearly important to consider for understanding larger scale habitat characteristics and should be examined to help identify larger-scale patch-size characteristics.

## Materials and Methods

### Study Area

**Figure 1:**
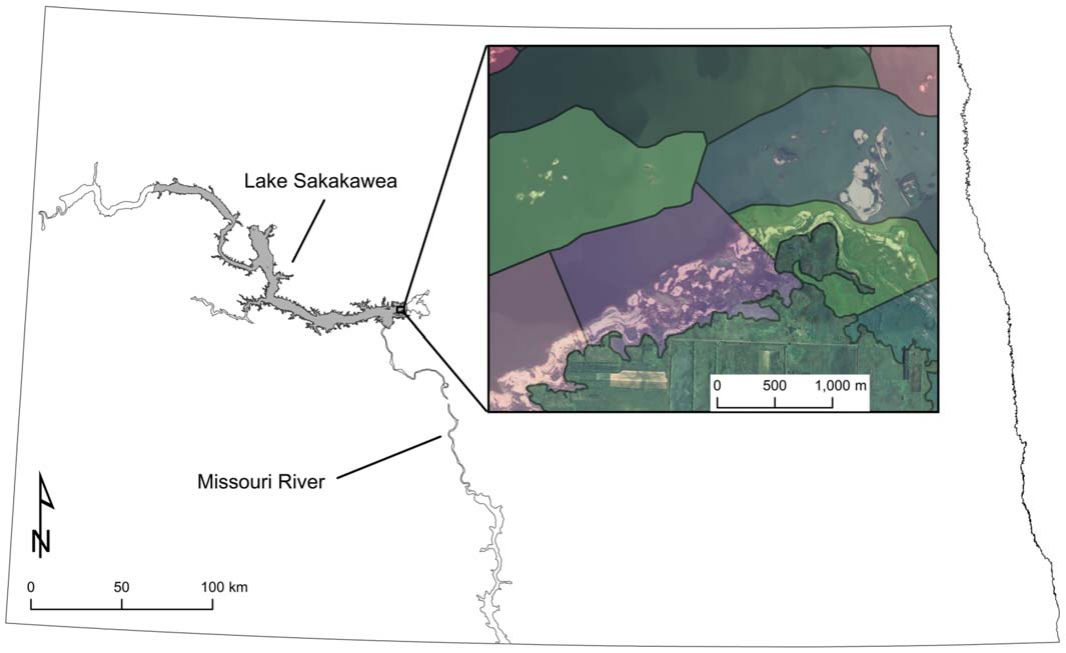
Lake Sakakawea study area. Map of North Dakota depicting our study area (shaded in gray) at Lake Sakakawea and an example of our segmentation of our study area.

**Figure 2:**
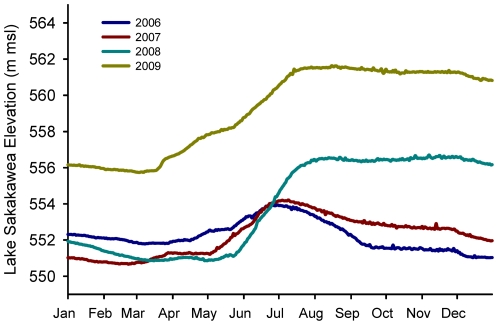
Water level at Lake Sakakawea during 2006–2009. Intra-annual fluctuations in water level at Lake Sakakawea during 2006–2009, adapted from [21].

SAK is a large (163,800 ha) main-stem reservoir located on the Missouri River in northwestern and central North Dakota (Figure 1) and is an important area for plovers; 43% of Missouri River adult plovers were observed there in 2005 (USACE, unpublished data). Lake level generally increases during early summer in response to Rocky Mountain snow melt (Figure 2) [21], [27]. Intra-annual increases in SAK water level occur at approximately the same time plovers begin initiating nests [27], which potentially puts nests at risk of inundation if they are initiated at low elevation.

Our study area included all shoreline and island habitats of SAK, ranging from Garrison Dam near Riverdale, ND to White Tail Bay, ND [37]. Each year the lower boundary of our study area was the shoreline and the upper boundary was at maximum flood level of the reservoir (565 m MSL; Figure 1). The reservoir shoreline is irregular, dissected, and consists of numerous substrates, slopes, and aspects. The distribution and area of these features vary annually as lake elevation changes in response to precipitation, melt of Rocky Mountain snowpack, and releases from Garrison and Fort Peck dams. Pre-flooding topography and hydrologic processes have created diverse habitat conditions including wide beaches where shoreline slopes are gradual, narrow beaches with a terracing pattern of slopes, and cut banks or bluffs of varying elevations. Sediments from a variety of parent materials, including sandstone, granite, and coal, have been exposed on the reservoir landscape providing variability of background colorations in nesting substrate. A wide range of substrate compositions is available on beaches in various distributions of grain sizes [16]. Throughout the reservoir larger sediments, such as gravel, have been deposited in narrow bands (1–3 m) dispersed on the shoreline slopes; however, larger expanses of gravel are situated in flatter areas previously exposed to wave action. Fluctuations in water level scour away terrestrial vegetation, thus when water recedes bare substrate is exposed. Newly exposed substrate may become vegetated rapidly in places where soil conditions are suitable or more slowly in areas where finer substrates and organics are eroded away. Dominant plant species typically were early successional or disturbance-adapted species including: cottonwood (*Populus deltoides*), sandbar willow (*Salix interior*), Canada thistle (*Cirsium arvense*), kochia (*Kochia scoparia*), meadow foxtail (*Alopecurus pratensis*), Russian thistle (*Salsola traqus*), sweet clover (*Melitotus spp.*), and various grass species.

### Sampling Design and Allocation

We used a stratified-random sampling design based on historic plover nest densities (USACE, unpublished data) to allocate our survey efforts. We used 2004 National Agricultural Imagery Program photos to delineate the shoreline of SAK. The shoreline was divided into segments of ∼2 km in length (Figure 1). Segments were classified into strata based on counts of nests from 1998 to 2005, and defined as low (<2 nests), medium (2–9 nests), and high (>9 nests) density. Sampling intensity in each stratum was determined using Neyman allocation [38] and a total of 37 different 2-km segments were randomly selected for surveys in years 2006–2009. We selected fewer segments in 2006, but in 2009 we selected 7 additional segments because increasing water level inundated 1 and markedly reduced available habitat on other segments (Table 4).

**Table 4:** Numbers of 2-km segments of shoreline surveyed within high, medium, and low nesting density strata on Lake Sakakawea, North Dakota, during summers 2006–2009.

Year	Total	High	Medium	Low
2006	17	7	5	5
2007	30	18	7	5
2008	30	18	7	5
2009	35	24	6	5

We systematically searched study segments for nests every 2 to 3 days throughout the nesting season (April–July). During systematic searches we searched for nest bowls with eggs and adult behavior consistent with nest defense. Upon discovery of a nest, we recorded a GPS location (post-processed differential correction; Trimble® model GeoXT, Trimble Navigation Limited, Sunnyvale, California) and floated eggs to estimate incubation stage [39]. We estimated nest initiation date by subtracting the number of days incubated and 2 days for each egg from the date of discovery.

### Nest-site Characteristics

We measured habitat characteristics at the nest site (1-m^2^ quadrat centered on the nest) and at 3-m and 10-m radiuses from the nest (hereafter nest areas). Nest areas consisted of 4 habitat measurements using the 1-m^2^ quadrat in each cardinal direction (n = 4 for each distance). We recorded all measurements upon nest discovery or on the second visit. We also collected habitat characteristics at 2 randomly selected reference sites for every nest (hereafter, random site) using the same methods for quadrat placements (1-m^2^ quadrat at the random site and 4 quadrats for each 3-m and 10-m area). Random sites represented unused available habitat within the nesting territory and we limited them to be within a 100- to 150-m radius of the nest. We used 100 m as the minimum distance between a nest and random point to minimize disturbance to the attending adult plover. However, on small islands (<∼3 ha), we selected random sites from within 10 to 100 m of the nest site. The distances we used to define plover territories were consistent with those reported for plovers nesting in other breeding areas at similar densities [40], [41]. If a random site contained >50% vegetation cover, or had a slope >100% (>45°), a new random site was selected. If a random site was within 3 m of a different plover nest, determined using GIS software after the field season, it was deleted (n = 6). We collected position data for each location where habitat data were collected.

We measured the elevation of each sample point using a 1) rotating laser level (LaserMark™ LMH-GR, CST/berger, Watseka, Illinois) relative to a nearby temporary elevation benchmark or 2) real-time kinematic survey (Trimble GPS model 5800 and 5700); benchmark elevations were measured annually using survey-grade GPS equipment (Fast-static data collection >120 min, National Geodetic Survey OPUS-static processing [42], Trimble® GPS model 5800 and 5700). At each nest and random site we measured elevation at 1 m distance upslope from the site, to calculate slope within 1 m of the site. At nest and random sites we also measured shortest distance to the shoreline of SAK using a laser range finder (Bushnell Elite 1500, Overland Park, Kansas, and Opti-Logic 800XL, Tullahoma, Tennessee).

Within each quadrat we collected a suite of habitat variables reflecting vegetation abundance and cover, substrate composition, and debris cover. We used a modified Daubenmire [43] classification for visual coverage estimations of vegetation, substrate and debris variables. The classification was as follows: 0%, >0–5%, 6–15%, 16–30%, 31–45%, 46–70%, >70% [16]. Visual coverage estimates of vegetation were recorded separately for terrestrial woody, terrestrial herbaceous, and wetland herbaceous vegetation [16]. We estimated coverage of substrates for each of the following grain sizes: silt <0.125 mm, sand = 0.125–2 mm, small pebble = 2–4 mm, gravel = 4–64 mm, cobble = 64–256 mm, or boulder >256 mm [16]. We estimated visual coverage for small debris (<2-cm diameter objects), large debris (>2-cm), and leaf litter.

For our analysis, we converted all visual cover classes to the mid-point of each class percentage values [16]. We estimated the amount of bare-substrate obstruction by summing the mid-point percentages of all vegetation cover classes, leaf litter, and small debris. We further converted substrate type visual cover midpoints to a percent of total composition. We summarized cobble to presence (1) or absence (0) within the nest or random site because we assumed that the effect of cobble at nest sites would manifest at very small percent cover values. We included percent coverage of silt at the nest site in analyses because we assumed that silt would be associated with sites that are poorly drained and slightly lower than surrounding areas.

We calculated means of each habitat variable for 3-m and 10-m nest areas (n = 4 for each), except for percent cover of gravel at 10 m from the site. We excluded the minimum and maximum value for percent cover of gravel at 10 m because plovers appeared to nest in patchy gravel strips on the shoreline. We assumed that using the 2 middle points would better represent the matrix outside the patch because the patches are typically arranged in strips that might be sampled on a cardinal direction by chance.

We calculated maximum slope for 1-, 3-, and 10-m distances from each site. We calculated the relative elevation above the shoreline for each nest-site by subtracting mean reservoir pool elevation [21] on the estimated date of nest initiation from the elevation of the site (hereafter relative elevation). Relative elevation of random points was based on the initiation date of the corresponding nest. Because not all habitat evaluations were conducted at or near the nest initiation date, we included a supplemental linear distance to water measurement derived from the nest-site or random site location measured (ArcGIS 9.3 Near Proximity Analysis Tool [ESRI, Redlands, California]) to the shoreline determined using a digital elevation model (5 m post spacing, 1 m vertical accuracy RMSE, acquired in 2007, Intermap Technologies, Denver, Colorado) on the estimated nest initiation date. The supplemental measurement was used instead of the field measurement at nests or random points infrequently (n = 29, 8%), only when water level of SAK changed enough to have an appreciable influence on the location of the shoreline in most areas of the lake (>1.5 m change between nest initiation and habitat measurement).

### Statistical Analyses

We examined correlations among our covariates (PROC CORR) [44], and found little evidence of correlation (unless otherwise noted *r*<0.33). However, relative elevation and distance to shoreline; slope at site and slope at 3 m; and slope at 3 m and slope at 10 m were correlated (*r* = 0.41–0.62). There were greater correlations within parameters measured at the site, 3 m, and 10 m (percent pebble, gravel, or bare-ground obstruction). Accordingly, we did not allow any measurements taken from the site and 3 m or 3 m and 10 m of the same parameter to coexist in a model; this ensured correlations among covariates in any potential candidate model were below *r* = 0.64.

We examined factors that influence intra-territory nest site selection by plovers at Lake Sakakawea using multi-model inference [45] of conditional logistic regression models for grouped data (PROC LOGISTIC) [44]. We specified the identification number of the nest and associated random sites in the strata option and the response variable was whether or not a site was used for nesting. We used a binomial distribution and a logit link function in all models. We examined the following continuous variables 1) at the site: percent silt, percent pebbles, percent gravel, presence of cobble, percent bare-ground obstruction, slope, distance to shoreline, and relative elevation above pool; 2) at a 3 m radius from the site: percent pebble, percent gravel, percent bare-ground obstruction, and slope; and 3) at a radius of 10 m from the site: percent pebble, percent gravel, percent bare-ground obstruction, and slope. We pooled data over years because preliminary modeling indicated that the effect of year was not important despite our study occurring during periods of declining, stable, and increasing lake level. We did not include percent coverage of sand because sand generally was the uniform matrix within which all other substrate sizes were distributed; this exclusion avoided issues with the compositional nature of substrate variables.

We selected a relatively balanced *a priori* suite of 20 candidate models; each explanatory variable occurred in 7 or 8 candidate models. Candidate models ranged in complexity from 2 to 13 parameters; 50% of models had <6 parameters. Models were constructed to examine selection based on scales (e.g., site, 3 m, or 10 m), major characteristics (e.g., vegetation, substrates, or topography), and some combinations of characteristics and scale. For example, one model included all variables measured at the site, whereas another model included only substrate characteristics at the site.

We ranked models by Akaike's Information Criterion corrected for small sample size (AIC_c_), and calculated the relative importance of each explanatory variable by summing the Akaike weights (*w_i_*) for all models containing the variable [45]. We calculated model-averaged parameter estimates, lower and upper 95% confidence limits (LCL and UCL, respectively) for all covariates [45] using a SAS macro that was modified from Shaffer [46]. We calculated odds ratios for each continuous variable for which the 95% confidence limit of the model-averaged parameter estimate did not overlap zero. We used the first and third quartiles of the data from random sites as comparison levels for calculating odds ratios; we reversed the order of quartiles for variables with negative parameter estimates so that all odds ratios were >1. This procedure standardized our odds ratio estimates relative to the amount of variability present for each variable at SAK, allowing for comparisons of effect-size among variables; we referred to these estimates as standardized odds ratios.

Two similar models were the most parsimonious; they both contained the following effects: percent cover of silt and gravel at the site, presence of a cobble, relative elevation, slope at the site, percent bare-substrate obstruction and slope at 3 m, and percent cover of gravel at 10 m (Table 5). The model that received the most weight also included distance to shoreline (Table 5). All variables included in the 2 most parsimonious models had relative importance values of 1.00, distance to shoreline had a relative importance value of 0.64, and all other variables had a relative importance value of 0.00.

**Table 5:** The most supported 5 of 20 models evaluated to examine factors that influence intra-territory nest-site selection of Piping Plovers at Lake Sakakawea, North Dakota, including number of parameters (K), Akaike's Information Criterion for small sample size (AIC_c_), increase over the lowest AIC_c_ (ΔAIC_c_), and Akaike model weight (*w*_i_).

Model Structure (Parameters)	K	AIC_c_	ΔAIC_c_	*w*_i_
SILT^a^, GRAV^b^_S^c^, COB^d^, DIST^e^, RELEV^f^, SLOPE_S, VEG^g^_3^h^, SLOPE_3, GRAV_10^i^	9	449.9	0.0	0.64
SILT, GRAV_S, COB, RELEV, SLOPE_S, VEG_3, SLOPE_3, GRAV_10	8	451.1	1.2	0.36
SILT, PEB^j^_S, GRAV_S, COB, VEG_S, DIST, RELEV, SLOPE_S, SLOPE_3, PEB_10, GRAV_10, VEG_10, SLOPE_10	13	474.9	25.0	0.00
SILT, COB, DIST, RELEV, SLOPE_S, PEB_3, GRAV_3, VEG_3, SLOPE_3, SLOPE_10	10	478.6	28.7	0.00
SILT, GRAV_S, COB, RELEV, SLOPE_S, GRAV_10, VEG_10, SLOPE_10	8	478.6	28.7	0.00

a Percent coverage of silt in substrate at site.

b Percent coverage of gravel in substrate.

c Site measurement.

d Presence or absence of a cobble.

e Distance (m) to shoreline of Lake Sakakawea.

f Relative elevation (m) of the nest above the pool level at initiation.

g Percent bare substrate obstruction (vegetation+leaf litter+small debris).

h Mean of 4 measurements taken 3 m from the site.

i Mean of 4 measurements taken 10 m from the site.

j Percent coverage of pebble in substrate.
